# The Institutional Worker

**Published:** 1921-01-08

**Authors:** 


### INSTITUTIONAL WORKER

**Being a Special Supplement to "The Hospital"**

### OUR BUREAU OF INFORMATION.

### Rules for Correspondents

1. Every letter must be accompanied by the coupon to be cut from the back cover (inside page) of The Hospital, current issue, and must contain the name and address of the correspondent with pseudonym for publication if desired. Replies by post cannot be given save under exceptional circumstances at the Editor's discretion.

2. Letters from Approved Homes in reply to special needs published in the Bureau should state terms and full particulars, and be sent prepaid under cover to the Editor of the Bureau with name written across coupon for identification.

3. Proprietors of Homes which have not yet been entered on the List of Approved Homes, but have spare accommodation likely to suit special needs, are invited to write for an application form for registration. The fee for registration, which includes two announcements of the Home in the Bureau and other privileges, is 10s.

4. All communications to be addressed to the Editor of The Hospital, 28 Southampton Street, Strand. London, W.C. 2, and marked "Bureau of Information."

### INSTITUTION FACTS AND FIGURES.

### HOTELS AND CLUBS FOR PROFESSIONAL WOMEN.

The solution of the housing problem is still a long way off, notwithstanding the numerous schemes now in progress to relieve the situation. The lack of suitable accommodation is a source of discomfort to perhaps hundreds of thousands of people throughout the country, but probably to none more than to that ever-increasing army of women workers in the large cities. Many of these have no homes other than those they can provide for themselves. At no time has there been adequate living accommodation of a character suitable for such workers, and to-day the condition of affairs is worse because, while the number of workers has increased enormously, social changes resulting from the war have tended to absorb accommodation previously available.

The ideal home for these workers is to be found in the well-managed residential club or hostel, for this provides at the same time privacy and companionship in cheerful surroundings, but the inadequacy of this kind of residence is demonstrated by the length of the waiting-lists. Even cheap boarding-houses are hard to find, and few of these provide the kind of home which is essential for health and happiness. The need has been by no means lost sight of, but the co-operative enterprise required for the establishment of residential clubs is lacking.

A year ago an editorial was published in the Journal of the Incorporated Society of Trained Masseuses (now known as the Chartered Society of Massage and Medical Gymnastics) with the object of stimulating interest in this domestic problem, and it produced an interesting letter, signed by several correspondents, outlining a scheme for the establishment of a residential club for masseuses. Certain friends interested in professional women had met and discussed the matter, and it was hoped that it might be possible to start the club this year. It is with regret we learn that the scheme has not matured, for it was an attractive and practicable one, and deserved success. The idea was, as soon as a suitable house should be found, to arrange it so as to suit all members, leaving some of the rooms unfurnished, so that those who possessed a little furniture might have the advantage of it; to furnish some of the rooms as bed-sitting-rooms, and to divide others into cubicles for those wishing to put up for a few nights only at a minimum of expense—the non-residential members to have the use of the public sitting-rooms and restaurant. It was proposed to form a limited company, so that the club might be run on business lines, and to invite masseuses to take shares and thus become co-operators.

Some useful and interesting facts which illustrate the possibilities in this direction may be gleaned from the working of the admirably administered Government hotels for women at Washington. These hotels are operated by the United States Housing Co-operation, and came into existence as a result of the recommendation of the Council of National Defence. During the war the Housing Bureau had under consideration a large number of schemes planned to provide for 250,000 persons, but at the signing of the armistice the number was considerably reduced.

The provision at Washington includes two groups containing twelve hotels, accommodating 1,950 women. In each there is an administration hall, a recreation-hall, two dining-rooms, an infirmary, a power house, a laundry, a refrigerating plant, and a storehouse. Each house or hotel is planned on the "U" shape, which provides two wings connected by a roof garden, accommodating 162 women in 122 single rooms, and only twenty double rooms. Each room is furnished simply but artistically with a bed, bureau, two chairs, a writing-desk, a nice clothes-press, and a cedar utility closet. There are five social rooms, dining-room, card-room, and reception rooms for the entertainment of guests. The terms of admission merely require the applicants to be labouring women and federal employees.

The management of the hotels is especially commendable. The only restriction—and that is one necessary for the protection of the property and the safety and health of the guests—is that at eleven o'clock the house closes for outside guests. Watchwomen patrol the corridors at night. They are of double service, for they not only protect against fire, but they are a strong contributing force to the happiness of the guests. If anyone is ill at night they call assistance or telephone for the nurse or doctor. These watchwomen are specially commissioned; they belong to the police force, and have police power.

The guests pay twenty dollars a month for their rooms, and twenty-five dollars a month for their meals. A moderate sum, though somewhat more than many of the hospital and health workers could afford to pay.

The government of these hotels has now passed into the hands of women, the management including a staff of five. The administration has solved a problem which is a more complex one than the hotel problem; it has succeeded in providing homes adapted to the needs of independent business women who want independence with an opportunity for social life, together with the protection of a well-conducted home.

That the accommodation and service are excellent there can be no doubt, nor is the social side overlooked. The recreation-hall plays a large part in the life of the guests. They have a course in gymnastics, in folk dance, in interpretive and social dancing. The infirmary gives three kinds of service—house service, where the nurse goes to the room or to the different hotels to care for the patients; the hospital service, where patients are taken into hospital, and the dispensary service.

That the scheme has justified itself is evidenced by the fact that at the

(*Continued on p. 2, col. 3.*)

### Question for January.

CHECKS ON WASTE.

Describe methods for keeping a check on the use of drugs and dressings in the hospital wards and operating-theatre with a view to prevent waste.

**(A minimum payment of 10s. 6d. will be made for each published answer.)**

RULES.

**The following rules must be observed :—**

**1. Contributions must be written on one side of the paper. Conciseness and terseness are desirable features. The MS. must bear the name and address of the sender and be accompanied by coupon to be cut from the back cover (inside page) of the current issue of The Hospital. A pseudonym must be chosen if the name is not to be published.**

**2. Contributions must be addressed to the Editor of The Hospital Institutional Worker Supplement, 28 & 29 Southampton Street, Strand, London, W.C. 2, should reach him before the end of the current month, and be marked in the left-hand corner "Facts and Figures."**

### QUESTIONS INVITED.

**In connection with our Question Box, we offer every month five shillings for the best question sent in for consideration. The questions may be on any institutional subject and concern any department, from the secretary's to the porter's, or the matron's to the domestic staff; the one essential is that they must be practical and deal with points that have a definite relation to hospital or institutional administration, upkeep, finance, or management. Points relating to artificers' work, housekeeping, the laundry, out-patients, and other practical matters will be welcome. It is hoped that thus, when difficult or doubtful points arise in the course of their work, institutional workers will be encouraged to put them in the form of a question and send them to the Editor, The Hospital Bureau of Information, 28 Southampton Street, Strand, W.C. 2, marked "Question Box."**

**The best questions will be published in due course and our readers will be given the opportunity of answering them. Thus all institutional workers may be able to co-operate with the view of helping the work and smoothing the difficulties of each other. In short, we want the Question Box of the *Institutional Worker* to be freely used by every worker habitually.**

### ENQUIRIES AND ANSWERS.

### *THE SICK AND IN NEED.*

#### Hospital for Baby suffering from Tuberculous Joint.

Write to the Medical Officer of Health for Liverpool or to the Liverpool Child Welfare Association and inquire if they can recommend your baby for admission into the Liverpool Babies' Hospital, Leasowe, Cheshire. Give full details of the case when writing. Parents pay a small weekly sum towards maintenance, according to their means.—Anxious.

#### Schools for Instructing Allied Children.

We suggest that you write for full information regarding the instruction of blind and partially blind children to the Education Officer, London County Council Education Offices, Victoria Embankment, W.C. 4. The London County Council have schools, both day and residential, for the instruction of blind and partially blind children. No fee is charged for education, but parents are required to contribute according to their means towards the maintenance of cases taken into residence. The schools are situated in various districts in London.—Parent.

#### Convalescent Nursing Home for Child.

The Children's Rest Convalescent Nursing Home, Roehampton, which is incorporated with the Friends of the Poor, 40-42 Ebury Street, S.W. 1, receives girls between the ages of three and thirteen. Admission is by payment of 10s. per week. Both helpless cases and open wounds are taken. The Abraham Ormerod Convalescent Home for Children, St. Anne's-on-the-Sea, also receives cases with open wounds. Admission is free with subscriber's letter for three weeks, or without letter £1 10s. for three weeks. Write to the Secretary, Church Extension Association, 27 Kilburn Park Road, London, N.W., for full particulars.—E. M. C.

### *MISCELLANEOUS*

#### How to Help the Hospitals.

S. H. Rylett, of 2 Southampton Street, Strand, W.C. 2, has a large assortment of attaché, writing, blouse, music, and other cases. He has issued a circular stating that he will give to the hospitals in the name of the purchaser 25 per cent. of the purchase money paid for articles bought from him. We shall be interested to hear from Mr. Rylett the result of his offer.

#### Smart-Bristow Coil.

A Smart-Bristow coil as supplied by Messrs. Watson & Sons, Ltd., Scenic House, Parker Street, Kingsway, London, W.C. 2, costs about £6 15s. This is worked from two dry cells. A resistance to work the coil from the continuous current main costs £1. The instrument is specially constructed for producing painless muscular contraction.—Masseur.

### *EMPLOYMENT AND TRAINING.*

#### Nurse Desirous of Settling in South Africa.

Write for advice to the Secretary of the South African Committee of the Society for Overseas Settlement of British Women, Hotel Windsor, S.W. The Society is working in conjunction with the Government to enable them to meet, select suitable candidates for suitable positions, and make all other necessary arrangements for the comfort of women landing in South Africa. The Society issue a strong warning to those women intending to go out there not to go alone or without assured employment.—Discontent.

#### Maternity Nursing.

Write to the Matron of The Mothers' Hospital, 153-163 Lower Clapton Road, E. 5, and ask if she has a vacancy for a probationer. You can obtain a six months' course in midwifery at this institution. The hospital contains seventy-five beds.—Pegs.

#### Instruction in the Nursing of Cancer Patients

You can obtain the instruction you require at the Middlesex Hospital as you have already obtained your general certificate of nursing. The instruction covers a period of twelve months, after which time a special certificate is granted. The commencing salary is £40 per annum, together with board, residence, part uniform, and laundry. Write to the Lady Superintendent and ask if she has a vacancy.—Nurse Edith.

#### Private Nursing Institution for Children.

There is a private nursing institution in connection with the Victoria Hospital for Children, Tite Street, Chelsea, S.W. We do not know if nurses other than those trained at the Victoria Hospital are attached to the private nursing staff, but write to the matron and inquire. The nurses receive a salary of £40 per annum, with 12½ to 25 per cent. on earnings.—Kiddie.

### HOTELS AND CLUBS FOR PROFESSIONAL WOMEN.

(*Continued from p. 1.*)

present time there are 1,400 applicants for rooms on the waiting-list.

A comparison between the advantages which a well-conducted home of this character offers and the deadly existence in a drab London boarding-house should be a sufficient stimulus to co-operative enterprise to provide for one of the greatest needs of the day. The matter is one which might readily be dealt with by the workers themselves. A common platform from which to voice the needs and to form the nucleus of an organisation is available, and it remains only for public spirit and persistent enthusiasm to carry the idea to a material issue.

